# Brain metastases and lung cancer: molecular biology, natural history, prediction of response and efficacy of immunotherapy

**DOI:** 10.3389/fimmu.2023.1297988

**Published:** 2024-01-12

**Authors:** Maria Sereno, Irene Hernandez de Córdoba, Gerardo Gutiérrez-Gutiérrez, Enrique Casado

**Affiliations:** ^1^ Medical Oncology Department, Infanta Sofía University Hospital, Madrid, Spain; ^2^ European University of Madrid, Madrid, Spain; ^3^ Fundación para la Innovación e Investigación Biomédica (FIIB) Hospital Universitario Infanta Sofía (HUIS) Hospital de Henares (HHEN), Madrid, Spain; ^4^ Instituto Madrileño Investigación Estudios Avanzados (IMDEA), Precision Nutrition and Cancer Program, Clinical Oncology Group, IMDEA Food Institute, CEI Universidad Autónoma de Madrid (UAM), Consejo Superior de Investigaciones Científicas (CSIC), Madrid, Spain; ^5^ Neurology Department, Infanta Sofía University Hospital, Madrid, Spain

**Keywords:** brain metastasis, organotropism, immunotherapy, abscopal effect, intracranial activity

## Abstract

Brain metastases stemming from lung cancer represent a common and challenging complication that significantly impacts patients’ overall health. The migration of these cancerous cells from lung lesions to the central nervous system is facilitated by diverse molecular changes and a specific environment that supports their affinity for neural tissues. The advent of immunotherapy and its varied combinations in non-small cell lung cancer has notably improved patient survival rates, even in cases involving brain metastases. These therapies exhibit enhanced penetration into the central nervous system compared to traditional chemotherapy. This review outlines the molecular mechanisms underlying the development of brain metastases in lung cancer and explores the efficacy of novel immunotherapy approaches and their combinations

## 1 Introduction

Lung cancer (LC) stands as the leading cause of cancer-related deaths, with brain involvement accounting for a significant portion of this mortality rate. LC contributes to approximately 50% of all brain metastases (BM) cases following melanoma (1, 2). The incidence of this complication is notably prominent in tumors featuring activating mutations (EGFR, ALK, and ROS-1). However, in patients without these molecular alterations, the incidence of brain involvement varies, occurring both at diagnosis and at different stages during the progression of their disease (3). The development of targeted therapies and immunotherapy has substantially enhanced the prognosis of non-small cell lung cancer (NSCLC) with brain metastases. This progress is particularly significant for patients with molecular alterations due to the emergence of increasingly effective treatments targeting the central nervous system (CNS). Notably, improvements in the prognosis of brain disease have extended beyond cases related to EGFR or ALK mutations. Even in patients lacking activating mutations, advancements in new immunotherapy regimens and combinations of chemotherapy and immunotherapy, including dual immunotherapy, have contributed to better outcomes (4). In addition, there is an increase of the rate of BM diagnosis. Several factors may have contributed: a longer survival in LC: advances in neuroimaging techniques; a rise in advanced cancer stages as well as a growth of the aging population (5).

It is evident that brain involvement has an impact on the prognosis and quality of life (QoL) of these patients. Multiple studies have attempted to identify high-risk individuals. Most clearly defined risk factors associated to BM development are: age of ≤ 60 years, non-squamous cell carcinoma, bulky mediastinal lymph nodes (> 2 cm) (6). In addition, other prognosis factors are classically associated a better survival: Karnofsky performance status (KPS) > 70%, the absence of extracranial metastases and control of the primary tumor (7).

Because is a relevant event in LC outcome, It is important to know the main molecular mechanisms underlying the development of BM and how this lesions could produce the invasion and dissemination in the central nervous system (CNS). Different factors such as loss of integrity of the blood-brain barrier (BBB), recent description of stem cells, interactions with the tumor microenvironment, loss of cell adhesion, the role of non-coding RNAs, etc., influence their appearance (8).

Here, we will describe the role of immunotherapy (IT) in BM treatment, trying to review the results of the main studies in the subgroup of patients with CNS involvement and to identify predictors and prognostic factors of the evolution of these patients.

## 2 Brain organotropism in lung cancer

Tumors originating in various organs exhibit a tendency to metastasize to specific organs, a phenomenon referred to as ‘organotropism.’ This process is believed to be governed by a range of factors, encompassing circulatory patterns, anatomical proximity, the metastatic environment, and intrinsic characteristics of the tumor cells (9). For tumor cells to infiltrate the central nervous system (CNS), they encounter the blood-bhrain barrier (BBB), where the most of them typicallyperish upon crossing. Consequently, those metastatic cells that breach this barrier need to acquire intricate mechanisms empowering them to invade, migrate and proliferate within the brain´s tissue, leading to the formation of macro-metastases. This demands the development of self-sufficiency, interactions within the brain´s tissue, leading to the formation of macro-metastases. This demands the development of self-sufficiency, interactions within the microenvironment, and the generation of the growth factors that spur tumor progression within this unique context (9). These circulating tumor cells responsible for dissemination, once transported through the circulatory system, extravasate and invade the parenchyma of the different tissues, forming niches that give rise to micro-metastases (10). Within these heterogeneous cell nests, cells with self-renewal or stem-like capacity, called cancer stem cells (CSCs), can inhabit, with the capacity to differentiate into different tissues and adapt to different microenvironment (11). Epithelial-mesenchymal transition (EMT) is an evolutionary-conserved process that is considered crucial for physiological embryonic development. However, recent data has indicated that EMT is also implicated in the processes of cancer progression and metastases. In this transition, cancer cells acquire characteristics of stem cells such as self-renewal and differentiation, losing their polarity and cell-cell adhesion structures, rearrange their cytoskeleton and become motile and resistant to apoptosis permitting seed new tumors, called also tumor initiating cells. Some markers of CSC have been identified: CD44, CD24, CD34, CD133 and CD117, as well as aldehyde dehydrogenase 1A1 (ALDH1A1) (12). Therefore, there is evidence that the role of EMT is fundamental in carrying out the BM development, since one of the main characteristics of metastatic brain lesions is the loss of epithelial attributes. The intercellular adhesion of this type of cells is established through E-Cadherin. Several authors have demonstrated a greater loss of E-cadherin in patients with NSCLC and BM compared to those without (13). Other markers frequently over-expressed in patients with BM are N-cadherin and vimentin, involved in migration and adhesion mechanisms with prognostic value associated and related to an increased risk of relapse according to some authors (14). Loss of EMT in the context of metastatic development also induces tumor stromal degradation through upregulation of matrix metalloproteases (MMPs) and plasminogen activators (PAs), favoring the development of an invasive phenotype (15). In this line, several authors have found elevated levels of MMP-9 in LC brain metastases compared to other tumors, suggesting a crucial role in the migration of tumor cells from the tumor circulation into the CNS through the blood-brain barrier (16).

On the other hand, several groups have also found that the CXCL12 levels and its receptor CXCR4 are higher in patients with brain metastasis compared to other tumors without, suggesting that the former have a phenotype more predisposed to cell migration since the interaction between both chemokines is associated with cell proliferation and migration (17). They also reported that CX3CR1-negative carcinomas preferentially metastasize to the brain, whereas CX3CR1-positive to other locations (18).

In addition to cytokines and markers of EMT loss, several growth factor pathways with protein kinase activity and their receptors have been implicated in the development of BM. Several RNA expression studies of BM in different tumors have shown that EGFR/ERK expression is elevated in tumors with brain involvement, and in the case of LC, it has even been found that the expression of the proteins of this pathway is higher in metastases compared to primary tumor (19). Activation of the c-MET-HIF pathway are over-expressed in BM also in CNMP patients mainly in a pathway of resistance to EGFR inhibitors in patients carrying EGFR activating mutations (20). On the other hand, the angiogenesis pathway is also of great importance in tumor invasion, including infiltration of the CNS. In this sense, the interaction between VEGF and its receptors induces angiogenesis by stimulating neo-vascularization and vascular permeability. Several studies have shown increased VEGF expression in patients with NSCLC with brain involvement, especially in adenocarcinoma. Thus, laboratory studies have shown in preclinical experiments that silencing VEGF reduced the incidence of BM (21). Several studies have demonstrated intracerebral activity of monoclonal antibodies that inhibit VEGF-R, such as Bevacizumab, given the overexpression of this pathway at this site (22).

In addition to the loss of EMT phenotype and overexpression of certain cytokines and receptor tyrosine kinase signaling pathways, other molecules have also been linked to intracerebral organotropism. MicroRNAs, non-coding RNA fragments containing between 18 and 24 nucleotides with the capacity to regulate post-transcriptional expression, have also been linked to intracerebral organotropism (23). It is known that almost half of the micro-RNAs described in the literature are associated with areas of the genome related to carcinogenesis and, as mentioned above, their inactivation or hyperactivation can regulate oncogenes or suppressor genes, giving a tumor phenotype. Regarding their role in the development of BM, it has been seen that in certain cases, there is a differential microRNA pattern between the primary tumor and BM, which leads us to believe that the latter give the tumor cells a specific tropism for brain involvement (24).

There are also studies implicating Mi-RNA dysregulation. Specifically, MiRNA-378 is differentially expressed in LC patients and BM compared to those without. This MiRNA-378 has been associated with increased cell migration leading to increased expression of metalloproteases (MMP-7, MMP-9) and pro-angiogenic factors (VEGF). Other authors attribute to Mi-RNA-200 dysregulation an important role in the metastasis development. In fact, elevated levels of this Mi-RNA-200 in cerebrospinal fluid are higher in patients with BM of mammary or pulmonary origin compared to those with primary tumors (25). Regarding EGFR disease, miRNA-197 and miRNA-184 upregulation has also been observed in this subpopulation of LC patients, concluding the potential value of these components as biomarkers associated to a higher risk of EGFR mutation. Finally, long non-coding RNAs (lncRNAs) is another class of non-coding proteins with an impact on tumor progression and migration (26). Shen et al. published that the expression of one of these RNA fragments, specifically MALAT1, has been associated with LC with an increased tendency to develop BM. MALAT1 has also been shown to induce up-regulation of the EMT phenotype in lung adenocarcinoma, to the extent that several authors have attributed to MALAT1 a potential value as a target for potential targeted therapies (27). **Table 1** collected all these alterations associated with the development of BM.

**Table 1:** Main molecular alterations present in brain metastases secondary to NSCLC.

Mechanism	Marker	Regulation
**EMT**	E-cadherin (13)	Loss expression
	N-cadherin (14)	Over-expression
	Vimentine (14)	Over-expression
	MMP-9 (15)	Over-expression
**Chemokines**	CXCL12-CXCR4 (17)	Over-expression
	CX3CR1 (18)	Loss expression
**Tyrosin Kinase Pathways**	EGFR/ERK (19)	Over-expression
	c-MET-HIF (20)	Over-expression
**Angiogenesis**	VEGF (21)	Over-expression
**Micro-RNAs**	MiRNA-378 (23, 24)	Over-expression
	Mi-RNA-200 (23, 24)	Over-expression
	miRNA-197 (23, 24)	Over-expression
	miRNA-184 (23, 24)	Over-expression
**Long non- coding RNA**	MALAT1 (25)	Expression

In conclusion, there are multiple mechanisms associated with tumor invasion of the CNS, which, as we have seen, include factors related to the loss of cell adhesion, interaction with the tumor microenvironment, cytokine activation, dysregulation of microRNAs, etc … inducing the development of metastasis in the brain. In this complex scenario, several studies have shown interesting activity of IT in BM. In the following, we will review different studies of IT and its efficacy in brain involvement.

## 3 Immunotherapy in CNMP with brain involvement without activating mutations

Since the advent of immune therapy (anti-PD-1 and PD-L1) in the NSCLC realm, numerous authors have assessed its impact on the central nervous system (CNS) both as a standalone treatment and in conjunction with chemotherapy within the brain, these innovative drugs with their distinct mode of action posed both a challenge in enhancing the efficacy of existing treatment strategies up to that point.

In the following, we will summarize the published data on the activity of IT, focusing in checkpoint inhibitors (ICIs) in patients with CNMP and brain involvement. We will first review the published data in monotherapy and then in combination.

### 3.1 Intracranial mono-immunotherapy activity in CNS metastasis

In the realm of immune therapy (IT), various studies have indicated that an increased presence of CD8+ T cells at the tumor site serves as a positive predictive factor for the response to anti PD-1 therapy. The intracranial response appears to be associated with the capability of cytotoxic T cells to migrate from the bloodstream to the brain, triggering T-cell activation in extracranial regions and lymph nodes, particularly in the deep cervical lymph nodes. Camy et al. reported a correlation between PD-L1 and CD-8+ expression in both brain metastases (BM) and corresponding primary tumors in 75% of patients who demonstrated an intracranial response (28).

Currently, we have three approved PD-1/PD-L1 inhibitors in patients with CNMP and PD-1 expression >50% (Pembrolizumab, Atezolizumab, Cemiplimab). We summarize the intracranial activity data from each of the approved drugs separately.

#### 3.1.1 Pembrolizumab

One of the first agents to evaluate the activity of IT in the CNS was Pembrolizumab, an anti-PD-1, has been approved as first-line setting for PDL1-positive advanced NSCLC patients according to KEYNOTE-0-24 study. In the main studies with Pembrolizumab, the rate of patients with stable or asymptomatic brain involvement was low and patients with untreated brain disease were excluded: KEYNOTE-010, 9. 18% (n = 28); in the KEYNOTE-024, 5.49% (n = 70); in the KEYNOTE-042, 12.2% (n = 15) in the KEYNOTE-021, 17.53% (n = 108) in the KEYNOTE-189, and 7.87% (n = 44) in the KEYNOTE-407. Data on the effect of anti-PD-L1/PD-1 on patients with non-treated or symptomatic brain disease are sparse. Perhaps the need for steroid administration in these studies limited their inclusion in the studies (29–31). A small subgroup analysis from the KEYNOTE-024 trial (Pembrolizumab versus Cisplatin-based chemotherapy) in 28 patients with BM, showed a prolonged PFS in patients who received Pembrolizumab (HR = 0.55, 95%CI 0.2–1.56) (32). A pool analysis of studies KEYNOTE-010, 001, 042 and 024, PD-L1 patients >1%, aimed to evaluate the role of pembrolizumab in monotherapy vs chemotherapy, a sub-analysis of 293 patients with BM, without differences in overall survival (OS) and progression free survival (PFS), although there was a higher response rate in patients with central involvement (30).

In addition to the Keynote studies, other authors have explored Pembrolizumab in BM setting. Goldberg et al, in a phase II study involving patients with BM from advanced NSCLC and melanoma, showed response rates of almost 30% in the overall population and 63% in PD-L1-positive. All patients with brain response also had systemic response and brain involvement. A subsequent update with a total of 42 patients observed similar response rates, but in this case, in PD-L1>1% patients (33).

A recent study investigating the actual pathway in stage IV NSCLC patients with brain involvement reiterated the efficacy of pembrolizumab as a standalone treatment in managing brain-ralated issues. Nonetheless, in ore debilitated patients with poorer ECOG status and symptomatic brain involvement, pembrolizumab administration did not demonstrate significant benefits (34).

Given that this drug was the first to be approved as first-line monotherapy in high-expressors, this is the setting in which we have the most data in the subgroup of patients with brain involvement. Data presented reflect the activity of this drug in BM in monotherapy vs. chemotherapy, with response rates, OS and PFS equivalent to that of patients without brain involvement. However, it should be noted that the patients included were stable and with asymptomatic brain involvement. Further studies in patients with untreated or symptomatic BM are crucial. **Table 2** collected different studies with Pembrolizumab in NSCLC.

**Table 2:** Summary of the studies with ICI monotherapy in patients with NSCLC and BM.

Trial	Patients in BM subgroup (n, %)	The patients excluded in BM	PD-L1 TPS %	Study Arms	OS m, HR PFS m, HR
Keynote 024 (32)	28 (9,18%)	Untreated or active	>50%	Pemb vs Platinum based CT	OS: NA; HR 0,73 (0,2-2,62) PFS: NA; HR NA
Keynote 042 (30)	70 (5,49%)	Untreated or active	> = 1%	Pemb vs Platinum based CT	OS: NA; HR NA PFS: NA; HR NA
Keynote 010 (30)	152 (14,7%)	Untreated or active	> = 1%	Pemb 2/10mg/m2 vs Docetaxel	OS: NA; HR NA PFS: NA; HR NA
Pool analysis 024, 001, 042 and 010 (30)	293 (9,24%)	Untreated or active	> = 1%	Pemb vs CT	OS: TPS>50% 19 ms vs 9,7 m HR 0,7 TPS>1% 13 m vs 10,3 HR 0,96 (NS) PFS: TPS>50% 4,1 m vs 4,6m HR 0,7 (NS) TPS >1% 2,3 m vs 5,2 m HR 0,96 (NS)
OAK (35)	118 (9,6 %)	Untreated or symptomatic	Any	Atz vs Docetaxel	OS: 16,1m vs 8,6 m; HR 0,59 PFS: NA; HR NA
Pool análisis OAK, FIR, POPPLAR, BRICH (36)	85 (10%)	Untreated or symptomatic	Any	Atz vs Docetaxel	OS: NA; HR NA PFS: NA; HR NA
CM-057 (37)	68 (11,68%)	Unstable or untreated	Any	Nivo vs Docetaxel	OS: 7,6m vs 7,3m; HR 1,04 NS
CM-017 (38)	17 (6,25%)	Unstable or untreated	Any	Nivo vs Docetaxel	OS: 4,9 m vs 3,8 m
Pool analysis 063, 017 and 057 (39)	88 (9,06%)	Unstable or untreated	Any	Nivo vs Docetaxel	OS: 8,4 m vs 6,2 m
Real life study (Gauvain)	63	No exclusion	Any	Nivolumab	OS 7,5 m PFS 3,5 m ORR intracranial 13%, DCR intracranial 51%
A french EAP (40)	197 (21%)	No exclusion	Any	Nivolumab	ORR intracranial 16%, DCR intracranial 17%
An italian EAP	409 (27, 5%)	Unstable or untreated	Any	Nivolumab	OS: 8,6 m PFS:3,6 m ORR intracranial 68%, DCR intracranial 40 %

BM, brain metastasis; Pemb, Pembrolizumab; Atz, Atezolizumab; CT, Chemotherapy; Nivo, Nivolumab; OS, Overall survival; PFS, Progression Free Survival; NA, non available; NS, Non significant.

#### 3.1.2 Atezolizumab

Atezolizumab is a humanized IgG1 anti-PD-L1 monoclonal antibody, approved for patients with metastatic NSCLC based on the results of the OAK study in platinum-pretreated patients. This study allowed the inclusion of patients with asymptomatic brain involvement, although again, under-represented (10% of the total population). Compared to Docetaxel, the results of Atezolizumab in patients with central involvement were significantly superior in terms of OS (20.1 vs. 11.9 months) (35). An OAK study update with 1225 intention-to-treat (ITT) patients confirmed the OS benefit of Atezolizumab versus Docetaxel in previously treated patients (36). A pool analysis including the main studies of Atezolizumab (FIR, OAK, PCD4989g, POPLAR and BIRCH) analyzed 79 patients with brain involvement, and although in terms of efficacy only those from the OAK study were analyzed where the benefit of Atezolizumab vs Docetaxel was confirmed as discussed above, in terms of safety there was no difference in toxicity between patients with or without brain involvement, with more neurological symptoms in the former (41). The IM power110 study, which gave the first-line indication to a high-expressing population, demonstrated a significant benefit in OS benefit with Atezolizumab versus chemotherapy. Although this study allowed patients inclusion with asymptomatic or treated brain involvement, we do not have data on the evolution of these patients (42).

#### 3.1.3 Cemiplimab

This drug is a humanized antibody with anti-PD-1 activity, the latest drug approved for first-line treatment in patients NSCLC with high-expressing. The EMPOWER-Lung study, which gave approval for this indication to Cemiplimab, included 83 patients with stable brain involvement, showing that in the Cemiplimab arm vs chemotherapy, the OS and PFS data were superior: OS (HR for OS: 0.17; 0.04-0.76) and PFS (HR for PFS: 0.45; 0.04-0.92) as had been shown in the non-brain population. 17; 0.04-0.76) and PFS (HR for PFS: 0.45; 0.22-0.92) as had been shown in the non-brain population (43). According to new data from a *post-hoc* analysis, at a median follow-up of 33.3 months (range 24.0–50.3) among patients with clinically stable brain metastases at randomisation, Cemiplimab (n=34) prolonged median OS (not estimable versus 20.7 months; hazard ratio [HR] 0.42; 95% confidence interval [CI] 0.20–0.87; p=0.0168) and median PFS (12.5 months versus 5.3 months; HR 0.34; 95% CI 0.18–0.63; p=0.0004) compared with CT (n=35). Cemiplimab also led to a higher ORR (55.9% versus 11.4%) (odds ratio 9.27; 95% CI 2.62–32.74; p=0.0002), a longer median duration of response (DoR) (31.7 months versus 12.5 months) and a lower rate of post-baseline brain-specific disease progression (14.7% versus 20.0%) than CT (37).

#### 3.1.4 Nivolumab

Another anti-PD-1 drug is Nivolumab, approved as monotherapy in platinum-pretreated patients according to CheckMate-017 and 057 studies results. In these studies, the percentage of patients with brain involvement was low, around 10%, and they had asymptomatic or controlled brain disease. Compared to the control arm, in this case Docetaxel, there was no difference in OS in the small subgroup of patients with central involvement (38, 40).

In another real-life analysis, this time with Nivolumab, another anti-PD-L1, which included a considerable number of patients, 409 patients with asymptomatic brain disease, Nivolumab monotherapy reported response rates of around 17% with a median OS of 8.6 months and median PFS was 3.0 months (39). In the French expanded use programme with Nivolumab (EAP), the brain-involved population was analyzed, with response and stabilization rates of around 48% and m OS was 6.6 months (95% CI: 3.8-8.3) (39). In an update analysis in CM227 study, 202 of 1739 randomized patients had baseline BM (nivolumab plus ipilimumab: 68; chemotherapy: 66). At 61.3 months’ minimum follow-up, nivolumab plus ipilimumab prolonged OS versus CT in patients with baseline BM (hazard ratio = 0.63; 95% confidence interval: 0.43-0.92) and in those without (hazard ratio = 0.76; 95% confidence interval: 0.66-0.87). In patients with baseline BM, 5-year systemic and intracranial progression-free survival rates were higher with nivolumab plus ipilimumab (12% and 16%, respectively) than CT (0% and 6%). Fewer patients with baseline brain metastases developed new brain lesions with Nivolumab plus Ipilimumab (4%) versus CT (20%). No new safety signals were observed (44).

Therefore, IT alone is an effective therapeutic option in patients with brain involvement, especially in high-expressor patients, where interesting response rates and median PFS rates have been found to be equivalent in many cases to those found in patients without brain involvement.

### 3.2 Intracranial double-immunotherapy activity in CNS metastasis

Another established strategy in the management of patients with advanced NSCLC is the combination of two ICI (anti-PD1/anti-PD-lL1 and anti-CTLA4). Both mechanisms of action are complementary to optimize the effect of cell-mediated immunity on tumor cells. CTLA-4 suppresses the immune response at the early T-cell proliferation stage, while PD-L1 at the late T-cell effect stage. This would indicate that inhibitors of the PD-L1/PD-1 axis and CTLA-4 would act at different stages of the process of anti-tumor immune activation. CTLA-4 inhibitors can increase infiltrating T-cell in tumor, while PD-1/PD-L1 inhibitors cannot. This makes it very attractive in the management of these type of patients, including brain affected patients, a situation in which the activity of this combination has also been explored (45).

The CheckMate-227 investigated the role of Ipilimumab combined Nivolumab in metastatic NSCLC patients in first-line treatment. In this study, 81 treated and asymptomatic BM patients were included, suggesting that the experimental combination group had a better OS than the CT arm (median OS: 16.8 vs 13.4 months) (46). CheckMate 817, a phase 3B study, evaluated flat-dose Nivolumab plus weight-based Ipilimumab in patients with metastatic NSCLC. Two cohorts were included in this study: an ECOG 2 and ECOG 0-1 cohorts, with comorbidities including untreated brain involvement. Although the primary objective of this study was toxicity, in this more fragile population the administration of double IT was associated with the development of acceptable and manageable toxicity in both cohorts (47). The CheckMate-9LA study analyzed the efficacy of the combination of CM 227 dual IT with two cycles of a platinum-based scheme versus platinum-based chemotherapy alone. In this trial CheckMate 9LA trial showed that the addition of 2 cycles of CT to Nivolumab plus Ipilimumab shows an advantage in OS compared to 4 courses of CT alone (15.6 versus 10.9 months, respectively) in overall population. Patients with asymptomatic or treated brain involvement were also included in this study. With a follow-up of 36 months, the efficacy of the combination at intracranial level in patients with pre-treated BM was quite significant, with even higher results in terms of OS and PFS than those observed in the overall population: OS: 19.3 versus 6.8 mo [0.45; 0.29-0.70] and PFS: 11.4 versus 4.6 m [0.42; 0.26-0.68] (48). In the same line of the CM 9LA study, the recently updated POSEIDON study with a similar design consisting of a combination of anti-PDL-1 and anti-CTLA-4 together with platinum-based chemotherapy vs chemotherapy alone, has recently been presented, with similar results to the CM9LA study, showing a benefit in favor of the combination in OS: median OS, 17. 2 months; 95% CI, 14.9-21.8) vs chemotherapy (median OS, 13.1 months; 95% CI, 10.6-15.1), with a risk for death reduction by 32% (HR, 0.68; 95% CI, 0.55-0.85). At 3 years, OS rates were 31.4% vs 17.3%, respectively. Population with asymptomatic or pre-treated brain involvement was also included, however to date no efficacy data are available for this combination in the subgroup of patients with central involvement (49).

Therefore, although data from large studies focusing on the analysis of the brain metastatic population are still lacking, it appears that this combination is an effective strategy in central involvement management in patients with advanced NSCLC. Future studies evaluating the role of the dual-immuno combination in the population with symptomatic or untreated brain metastases may not be available at the outset, but the incorporation of such regimens into our routine therapeutic arsenal may be the subject of real-life studies and will provide us with experience and information on potential efficacy in this setting as well as safety data.

### 3.3 Intracraneal chemotherapy-inmunotherapy combination activity in CNS metastasis

One of the most widespread strategies in the first-line treatment in advanced NSCLC is anti-PD-1/PD-L1 and CT combination. With the exception of the combination of Nivolumab with platinum-based CT, all other studies that have analyzed the benefit of the combination of these antibodies and chemotherapy have shown superiority of the combination over chemotherapy alone. Chemotherapy and ICIs synergies yield to NSCLC patients with BMs in prolonged survival, including PFS and OS. Data for the brain-affected population from the main studies of combination anti-PD-1/PD-L1 and CT are shown below. **Table 3** shows a summary of all combinations of chemo and ICI and their intracranial activity.

**Table 3:** Summary of the all the schemes of chemotherapy and Pembrolizumab combinations and their intracranial activity.

Study	Patients in BM subgroup	Patients with BM excluded	PDL-1 score	Arms	OS and PFS
Keynote 189 (49)	108 (17,5%)	Untreated or symptomatic	Any	Pembrolizumab + platin based chemotherapy vs platin based chemotherapy	OS: NA; HR 0,42 PFS: NA; HR 0,36
Keynote 021 (50)	15 (12%)	Untreated or symptomatic	Any	Pembrolizumab + platin based chemotherapy vs platin based chemotherapy	OS: NA; HR NA PFS:NA; HR NA
Keynote 407 (51)	44 (7,87%)	Untreated or symptomatic	Any	Pembrolizumab + Carbo-Paclitaxel/nab-paclitaxel vs Carbo-Paclitaxel/nab-paclitaxel	OS: NA; HR NA PFS:NA; HR NA
Pool analisys KN 021, 189 and 407 (51)	171 (13%)	Untreated or symptomatic	Any	Pembrolizumab + platin based chemotherapy vs platin based chemotherapy	OS: 18,8m vs 7,6m HR 0,48 PFS: 6,9m vs 4,1m HR 0,44

BM, Brain metastasis; OS, Overall survival; PFS, Progression free survival; NA, Not available.

#### 3.3.1 Pembrolizumab and chemotherapy

Different trials have noted that Pembrolizumab plus standard CT may improve the outcome of patients. The KEYNOTE-189 study, a pivotal study comparing Pembrolizumab in association with CT vs for first-line permited the approval in patients with advanced NSCLC with non-squamous histology, included 108 (17.53%) with asymptomatic or controlled brain metastases, a non-negligible number of patients considering the population included in the previously mentioned studies. In a recent update in this subgroup with central involvement, authors concluded that the mOS of the in Pembrolizumab group was significantly longer compared to placebo plus CT group (19.2 vs 7.5 months; HR = 0.41, 95% CI: 0.24-0.67) among BM patients (51). The Keynote 407 study, which focused on the squamous population with a design parallel to Keynote 189, did not analyze the evolution of patients with asymptomatic BM, who were also included in this study, perhaps justified by a smaller number of cases (in this histology the incidence of brain metastases was lower) (50). Powell et al. published a pooled analysis of the three trials (KEYNOTE-189, 021 and 407) including 1298 NSCLC patients, 171 with asymptomatic BM. In this population, HR for OS and PFS were 0.48 (95% CI: 0.32–0.70) and 0.44 (95% CI: 0.31–0.62), respectively, even better that those patients without (HR for OS and PFS were 0.63 (95% CI: 0.53–0.75) and 0.55 (95% CI: 0.48–0.63), respectively). From this pooled analysis looking at the role of the combination with Pembrolizumab, the activity of this intracerebral regimen is equally active, even superior to that seen in patients without CNS involvement (52). Afzal MZ et al. conducted a real-life study that included 54 advanced patients with non-squamous tumors, with a sub-analysis of a population of 18 patients with pre-treated or asymptomatic brain involvement. The study analyzed two cohorts, CT alone or CT and Pembrolizumab, noting that the results from this real-life study in terms of OS and PFS reproduced the data from the pivotal studies, both in patients with brain involvement and in those without central lesions (53).

#### 3.3.2 Atezolizumab and chemotherapy

Several studies in patients with advanced NSCLC: IMpower 130 (Atezolizumab + Carboplatin + Nab-paclitaxel; N = 451) and IMpower 150 (Atezolizumab + Carboplatin + Paclitaxel + Bevacizumab; N = 356), demonstrated the benefit associating Atezolizumab to platinum-based regimens, with an acceptable safety profile and a benefit in terms of OS and PFS. In IMpower 130 study, patients with treated asymptomatic CNS metastases were also eligible, but active or untreated CNS metastases, spinal cord compression, or leptomeningeal disease were ineligible. In the subgroups analysis, although an *a posteriori* review of patients with liver metastases was included, no specific analysis of the evolution of BM was done for the category of patients with liver metastases (54). In the IMPOWER 150 study (55), although it also allowed the inclusion of patients with treated or asymptomatic brain involvement, they found that the benefit in the population with central involvement was higher in ABCP regimen, that could delay the time to development of new BMs (HR=0.68) for ABCP versus BCP and 1.55 for ACP versus BCP). The ATEZO-BRAIN study, with a recent update at ASCO 2022, is a phase II study that examined the role of Atezolizumab in combination with CT in patients with untreated BM by evaluating the efficacy and safety of Atezolizumab plus Carboplatin with Pemetrexed every 3 weeks for 4-6 cycles, followed by maintenance with Pemetrexed plus Atezolizumab in stage IV non-squamous wild type NSCLC patients. This is the first study conducted specifically in patients with untreated brain involvement evaluating the role of IT in this setting. Out of 40 pts included in the study, 22 (55%) were receiving dexamethasone at baseline and 20 (50%) had positive expression of PD-L1. The authors found 40% ORR according to RANO criteria with 47% systemic responses. Median OS was 13.6 months (9.72 to not reached) and estimated 2-year OS rate (95% CI) was 30.5% (18.4 to 50.4). In PD-L1 positive patients, median OS was superior to PD-L1 negative patients (16.2 vs 10.7 months) but differences were not statistically significant due to the limited statistical power (HR = 0.99; 95% CI 0.35 to 2.12). Significant differences were also not observed in patients receiving dexamethasone vs. those not receiving (56).

## 4 Activating mutations and brain involvement: does IT play a role?

As discussed in previous paragraphs, standard CT does not adequately cross the BBB. However, tyrosine kinase inhibitors (TKIs), the first-line treatment of choice in patients with specific molecular alterations with specific targeted therapy, generally have high intracranial penetration rates. On the other hand, it should not be forgotten that this population has a high incidence of BM, which induces the need for therapies against this type of tumors to be especially effective at the intracranial level (57).

EGFR, the most frequently observed mutation in NSCLC, has several generations of targeted therapies. Osimertinib, a third-generation EGFR-TKI, has shown more potent efficacy in the treatment of BM in untreated EGFR-mutated NSCLC in FLAURA study. Other recent studies show that compared to first-generation EGFR-TKI, it is associated with longer OS in this group of patients (58). Tzu-Hsuan Chiu et al. obtained results in favor of the association of first-generation EGFR-TKI with bevacizumab in patients with brain metastases, improving treatment efficacy and OS (59).

It is classically known that EGFR or ALK disease is particularly resistant to the effect of ICIs. This same scenario is repeated in the context of brain involvement, where patients with these mutations are frequently associated with metastatic brain lesions. However, several studies have explored intracranial TI activity or combinations in patients carrying these activating mutations. Lu Shun et al, analyzed in a phase III study the combination of an anti-VEGF TKI (IBI1305) together with CT (Platinum-Pemetrexed) associated with an anti-PD-1, Sintilimab. The Chinese study included patients with NSCLC carrying EGFR mutations who had progressed to anti-EGFR. In this study, patients with BM were included (36% in each group), and a pre-established sub-analysis was planned in the BM subgroup. With 9-8 months of follow-up, it was observed that patients who had received the combination of Sintilimab-IBI305-CT had significantly longer median PFS than those in the CT alone group (median 6·9 months [95% CI 6·0–9·3] *vs* 4·3 months [4·1–5·4]; HR 0·46 [95% CI 0·34–0·64]; p<0·0001). The estimated PFS rate was 59% (95% CI 49–68) in the Sintilimab-IBI305- CT group versus 30% (22–39) in the CT alone group at 6 months, and 28% (18–39) versus 12% (7–20) at 12 months. The prespecified subgroup analysis showed that the HR for PFS favored patients receiving Sintilimab-IBI305-CT over those receiving CT alone across most subgroups, including patients with BM. The data from this study lack maturity, but all indications are that combinations with CT-anti-VEGF and IT may have some activity in BM patients (60). As previously mentioned, the IMPOWER 150 study also analyzes a combination strategy of IT-CT-anti-VEGF, although we do not have specific data in the brain-affected population in this study (55). In addition to EGFR, other molecular alterations, such as ALK or ROS-1 translocations, are also associated with a high incidence of BM. Drugs such as Lorlatinib, a third-generation inhibitor active on both targets, have demonstrated durable control of brain disease in patients with asymptomatic metastases without extracranial disease. However, evidence of intracerebral TI activity in these patients is scarce (61). Several combinations with antiangiogenic agents, CT/IT are being tested in this population, mainly in patients refractory to inhibitors, although we do not yet have data on their systemic or intracerebral efficacy as they are in the recruitment phase: NCT05266846II30: Chemotherapy + Bevacizumab + Pembrolizumab; NCT03991403 Phase III: Chemo + Bevacizumab + Atezolizumab; GFPC06-2018 NCT04042558 Phase II: Chemo + Bevacizumab + Atezolizumab; GFPC06-2018 NCT04042558 Phase II: Chemo + Atezolizumab +/− Bevacizumab; NCT05266846 Phase II: Chemo + Bevacizumab + Pembrolizumab).

Combinations with other anti-VEGF TKIs, such as levantinib, are being tested in combination with anti-PD-1 or other IT in patient’s refractory to ALK inhibitors and translocation, although mature data are not available as the trials are also in the recruitment phase: NCT0525296278II80 Phase IB: Lenvatinib + CT; NCT04989322 phase II CT + Pembrolizumab + Lenvatinib; NCT0525296278II80 IBI-322+ Lenvatinib + CT.

On the role of IT at systemic and intracerebral level with IT remains scarce and for most targeted therapies, we still have little clinical evidence of IT activity at systemic and intracerebral level. Published data come from clinical cases or case series. For example, in patients carrying mutations in K-RAS, which is frequently implicated in the oncogenesis of different tumors, including CNMP, Li Q et al. published that combinations of IT and CT were associated with increased response to treatment independently of PD-L1 and tumor mutation burden (TMB). The Cox multivariate analyzes showed that BM [hazard ratio (HR) =0.232, 95% CI: 0.102-0.530; P=0.001], TMB (HR =5.675, 95% CI: 1.948-16.535; P=0.001), K-ras mutation (HR =2.552, 95% CI: 1.141-5.708; P=0.023) were independent predictors of OS in patients treated with ICIs and platinum-based CT (62). In addition to combinations of IT and CT, for some years now we have had specific K-RAS inhibitors (Sotorasib, Agardasib), the first currently approved in those patients refractory to first-line platinum or combinations with IT (63).

Other molecular alterations such as BRAF, HER-2, RET, MET have recently approved targeted therapies in some cases even in first line. The activity of IT in this setting is more unpredictable. Efficacy data come from cases or case series. In the case of melanoma, ICI has shown significant intracerebral activity, especially in combination (anti-PD-1 + anti-CTLA-4) in patients with B-RAF mutations pre-treated with inhibitors (64). The evidence on LC in this setting is sparse.

In conclusion, in the setting of the specific molecular alterations in patients with advanced NSCLC, those with K-RAS mutations and brain involvement, we could say that they are the greatest beneficiaries of IT, especially in combination with CT.

## 5 Combinations with antiangiogenics

It is commonly believed that anti-angiogenic agents limit tumors growth by inhibiting the unregular vasculature of tumors. However, different studies have demonstrated that low dose of anti-angiogenic drugs could induce the normalization of abnormal tumor vascularization, decreasing hypoxia induced by tumor and increasing accessibility for immune cells (65, 66). Another approach in the management of advanced CNMP is combinations with anti-angiogenic drugs. Ma X et al. analyzed in a study with 85 patients the efficacy and safety of combined IT and antiangiogenic therapy for advanced NSCLC in the real world and observed a PFS 7.9 months, a median OS of 18.60 months and ORR of 32.9%. A sub-analysis in asymptomatic or treated brain involvement patients, was associated with worse PFS vs. the subgroup without brain involvement (p = 0.016). In addition, patients receiving IT combined with antiangiogenic therapy in second-line therapy had longer OS than those receiving IT in further lines (p = 0.039). AEs of different grades occurred in 92.9% (79/85) of NSCLC patients, most of which were mild grade 1/2 AEs (67).

Combination CT, antiangiogenic and anti-PD-L1 regimens are a therapeutic alternative in patients with EGFR mutations or ALK translocation. Efficacy data on OS and PFS in this subpopulation from the IMPOWER 150 study. In this study, patients were randomized 1:1:1 to receive Atezolizumab + Bevacizumab + Carboplatin/Paclitaxel (ABCP), Atezolizumab + Carboplatin/Paclitaxel (ACP), or Bevacizumab + Carboplatin/Paclitaxel (BCP). A significantly improved PFS and OS were observed in the ABCP group compared with the BCP group for metastatic non-squamous NSCLC. Outcomes from the latest IMpower150 exploratory analyzes in the subgroup with BMs, ABCP regimen could delay the time to development of new BMs (HR=0.68) for ABCP versus BCP and 1.55 for ACP versus BCP). In the BMs subgroup of Impower150 trial, the ABCP group had the highest incidence of Grade 3~4 trAEs among the three groups. Besides, treatment withdrawal due to AEs occurred in 42.9% of patients in the ABCP arm. On the plus side, there were no grade 5 AEs with ABCP (68). Experiences with the combination of anti-PDL-1 drugs have been published, supported by this rationale of enhancing the effect of IT by blocking tumor angiogenesis. A phase 1b trial assessed Sintilimab combined with Anlotinib (a multi-target tyrosine kinase inhibitor with anti-angiogenic action) in the frontline setting for advanced NSCLC. They included 4 patients with asymptomatic BM and all four involved patients with asymptomatic BMs at baseline achieved intracranial complete response, and three of them achieved overall partial response, indicating that Sintilimab plus Anlotinib had central synergistic effects (69).

One of the main concerns with the administration of antiangiogenic agents in patients with metastatic brain involvement is the potential risk of intracerebral bleeding. There is considerable evidence confirming the safety of these agents in this setting. Sandler et al, in a systematic review of the safety of VEGF inhibitors in patients with NSCLC and brain involvement, found no increased risk of intracerebral bleeding compared to patients who had not received anti-angiogenic therapy (70). Another anti-angiogenic drug widely used in lung cancer in combination with second-line CT (Docetaxel) is Nintedanib. The LUNG LUME 1 study was positive in demonstrating an increased benefit of the combination of CT and Nintedanib vs. CT alone in terms of OS and PFS. This study included almost 6% of patients with brain involvement in each arm, although there are no results on efficacy or toxicity in this specific population (71). A phase I safety study has been conducted in different tumor cohorts with the combination of Nintedanib and Pembrolizumab. Patients with stable BM were allowed, although there are no toxicity or efficacy data as this is a very limited population (72).

Therefore, published data on the brain activity of combinations with antiangiogenic drugs are promising, but specific comparative studies in this population are lacking to know the real value of this strategy.

## 6 Radiation and inmunotherapy

**Figure 1:**
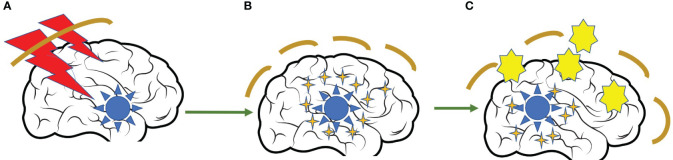
Abscopal effect of the radiation over tumor cells. **(A)** Intracranial metastasis treated with radiation therapy. **(B)** Rupture of the blood-brain barrier due to the effect of radiotherapy and release of antigens and cytokines into the circulation. **(C)** Facilitation of the penetration of dendritic cells into the CNS to promote LT activation and tumor destruction.

Radiation can damage the DNA of tumor cells, inducing the tumor cells death. These dead cells release antigens, which will promote DC-mediated antigen presentation. Eventually, it will activate and proliferate CD8^+^ tumor-specific T cells that can regulate the tumor microenvironment to promote the recruitment and infiltration of immune cells. These mechanisms provide a theoretical basis for a combination of radiotherapy (RT) and IT (73). The administration of RT in the context of LC is widespread. Almost all patients with advanced disease require treatment with palliative or symptomatic and sometimes radical radiotherapy at some point in their evolution. In patients with BM, the association of IT and RT may be of particular interest as both treatments would have a synergistic effect. Several studies have shown elevation in intracranial disease control, prolongation survival as well as improvement in neurocognitive function when both treatments are administered concurrently (74). The abscopal effect is a rare phenomenon but is well documented. Patients receiving RT to the brain or spine for metastatic disease and on immune checkpoint inhibitor therapy may benefit from a heightened immunologic response and regression of tumors distant to the irradiated site. Further investigation into the role of the blood–brain barrier (BBB), treatment strategies, and stratified patient populations most likely to benefit from the abscopal effect are necessary (75) (**Figure 1**).

A multicenter phase II trial examined the effect of combining nivolumab with RT. It included 26 patients (22 NSCLC, 12 cases with PD-L1≥50%) diagnosed of BMs with no previous treatment. Around 50% (12 patients) had high intracranial control with median intracranial PFS was 5.0 months and median OS was 14 months. In the sixth month, neurocognitive function showed potential improvements. Authors demonstrated in this research that, compared with RT alone, RT plus IT significantly improved local disease control and survival (74). Another analysis from the analysis of an American database of patients with NSCLC and brain involvement, the National Cancer Databank (NCDB), showed that the median OS of this population treated with IT and brain RT was significantly increased compared to those patients registered in the database who had received only RT without IT (76).

One of the main concerns with the combination of these strategies in patients with BM is the increased risk of radionecrosis, especially in high-dose-intensity treatments. This is a serious complication whose symptoms vary depending on size and location and range from nausea and vomiting, headache to neurological focality and seizures. Although it is a complication that can occur in patients with NSCLC, it has been seen more often in patients with BM from melanoma (77).

Despite the risk of this complication, studies examining the safety of co-administration of SBRT (stereotactic brain radiosurgery therapy) and IT have not demonstrated an increased incidence of radionecrosis after concomitant administration of both treatments (74). It is also unclear when is the optimal time to administer IT: before, during or after SBRT. According to the abscopal effect that could be triggered by the administration of RT, releasing neoantigens that favor immune activation and thus enhance IT, an ideal time could be after RT. Other authors advocate a concomitant administration of IT and RT (78). Jiang et al. conducted a retrospective study of patients receiving IT and stereotactic radiosurgery (SRS)/SRT comparing compared with the non-concurrent treatment methods, patients received concurrent treatment had a significant longer OS, reduced incidence of new BM lesions, increasing response rates with an acceptable safety profile (79). Hubbeling et al. conducted a similar study showing a benefit in favor of concomitant IT in patients with metastasis (77). However, not all studies confirm this synergy (80). Shanker et al. failed to demonstrate benefit in OS or CNS PFS between the SRS alone group and the concurrent SRS and ITs group in a study analyzing the effect of concomitant treatment (81). A meta-analysis showed that concurrent SRT with IT performed better OS than sequential therapy in the treatment of NSCLC patients with BMs (HR=0.39), but there were only two studies involved, these results should therefore be interpreted with caution (82). Another question that arises in this context is whether hypofractionation regimens might be superior to conventional regimens. Some authors argue that such regimens produce a superior activation of the anti-tumor immune response. However, other authors in retrospective studies have also found benefit in the administration of more palliative regimens, such as holocraneal RT in combination with IT. More prospective studies are needed to study the best timing of IT combined with RT and the optimal schedule (83).

Despite the theoretical benefit to our patients of combining IT and RT as outlined in the above studies, other authors found no benefit in the addition of RT to IT. A recent study compared the benefit of combining Pembrolizumab with holocraneal RT vs SBRT or Pembrolizumab alone in patients with LC and symptomatic BM. In this study, they found that administration of Pembrolizumab without RT shows similar results when it is associated with holocraneal RT or SBRT in intracranial response rate, so the authors suggest reserving RT for salvage treatment in case of non-response to IT (84).

## 7 New immunotherapy approaches in brain metastasis setting

In addition to ICIs, other new IT strategies have been evaluated in the context of BM in NSCLC. For the design of these new anti-tumor strategies, not only the tumor cell *per se* has been considered, but also the microenvironment in which this cell inhabits and thanks to which it subsists, proliferates and spreads. Thus, the brain microenvironment is emerging as a key player in defining the viability of tumor cells in this organ, and therapies targeting this microenvironment could become a reality in the future (85). In this section, we will review different innovative strategies in the field of IT and brain impairment.

### 7.1 miRNA-targeted therapies

The discovery of miRNAs in the tumor microenvironment and their relationship to cancer dissemination is a source of inspiration for new therapeutic approaches. Different therapeutic strategies such as miRNA replacements with oligonucleotides that silence oncogenic miRNA or restore tumor suppressor miRNA, have an increasing interest (86).

Basic research studies have shown for example that miRNA-768-3p drives K-RAS expression and is associated with resistance to anti-tumor treatment in BM; results of therapy with a miRNA-768-3p inhibitor *in vitro* reveal decreased miRNA-768-3p in patient BM compared to normal brain tissue and primary tumor tissue from the same patient (1).

The main limitation of these therapies, apart from the difficulty in identifying miRNAs, their inter-individual variability and the complexity of crossing the BBB, which seems to be being resolved with the use of lysosomes, is that miRNAs can target a multitude of genes and gene expression pathways, and therefore the effects of inhibiting or activating these pathways can affect the functioning of healthy cells, leading to toxicity and side effects (86).

### 7.2 Therapies aimed at EMT

Epithelial-mesenchymal transition (EMT) is a process that is part of normal embryonic development that it has recently been implicated in tumor progression and BM development, favoring cancer cells to acquire stem cell characteristics such as resistance to apoptosis (86). Over the last few years, targeted therapies for EMT have evolved significantly, although many of the studies are still in the recruitment phase (87).

### 7.3 Lymphocyte therapies

Tumor infiltrating lymphocytes (TILs) are a promising biomarker for assessing the tumor immune microenvironment and response to IT; their difficulty currently lies in the difficulty of their assessment. Ways of estimating TILs in clinical practice in different tumor types are being investigated (88). The amount of TILs in BM has been shown to correlate with OS and the amount of peri-tumoral oedema, identifying the immune system as a potential biomarker for cancer patients with CNS involvement, although further studies are needed to validate these findings (89).

Recent research has used oncolytic viruses as a potential therapy for BM. These are micro-organisms that target the tumor cell which, once infected, is destroyed by the patient’s immune system, secondarily stimulating the production of IFN and PDL1. This makes PDL1-targeted therapies more effective. The limitation of this treatment is the risk of viral infection by infiltration of non-cancerous cells (90).

On the other hand, chimeric antigen receptor-modified T-cell therapy (T- CAR), TIL therapy and T-cell receptor (TCR) therapy, are beginning to be used in the context of BM and positive results have been obtained in some tumors. Other authors used an optimized T cell, in their case HER2-CAR T cells for the treatment of brain metastases from breast cancer, with favorable results *in vitro*. No data on studies in the context of LC with BM have been found (91, 92).

## 8 Conclusion

Brain involvement is a therapeutic challenge in the management of patients with NSCLC, especially in patients who do not carry activating mutations amenable to targeted therapies with high rates of intracerebral penetration. It is in this subgroup of patients that IT administration is likely to have poorer outcomes than tyrosine kinase inhibitors.

IT is a therapeutic option of great interest in other patients, and in this review, we have shown studies with this type of treatment administered in monotherapy or in combination, with particularly interesting results.

The absence of predictors of extracranial response to IT, beyond PD-L1, TMB, TILs, can be extrapolated to intracranial response, so more studies are needed in this context, and ideally in the setting of brain involvement, to optimize patient selection.

IT in monotherapy, combinations with CT, double IT with or without CT or with anti-angiogenics have demonstrated intracranial activity, with results equivalent in OS to those observed in patients without brain involvement, and in some cases even superior.

The administration of RT and IT is becoming increasingly widespread given the biological enhancement effect of the latter on IT, favoring the abscopal effect and promoting inflammation and lymphocyte mobilization as an IT-enhancing strategy. Evidence is lacking to decide the safest sequence and doses to avoid radionecrosis.

New IT strategies offer hope for those patients most resistant to anti-PD-L1/PD-1. Multiple studies are evaluating different combinations in order to optimize control of these patients.

## Author contributions

MS: Conceptualization, Investigation, Supervision, Writing – original draft, Writing – review & editing. IH: Writing – original draft, Writing – review & editing. GG-G: Writing – review & editing. EC: Writing – review & editing.
